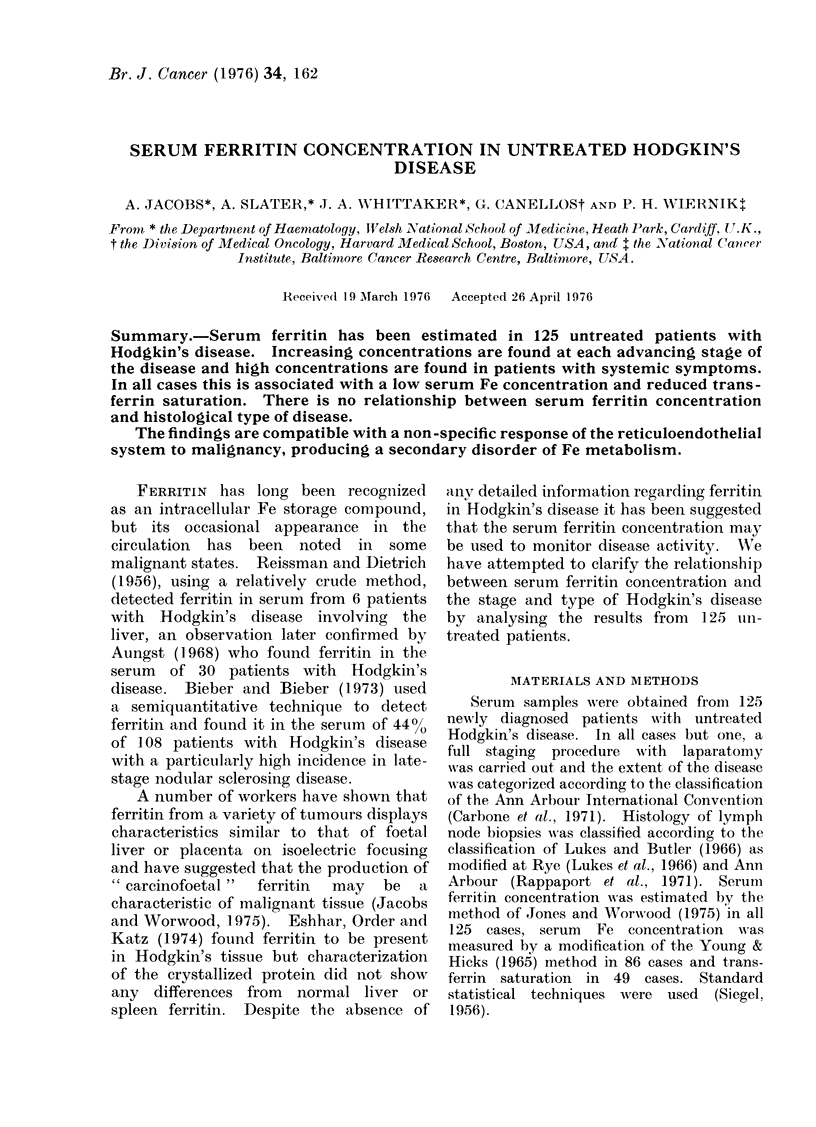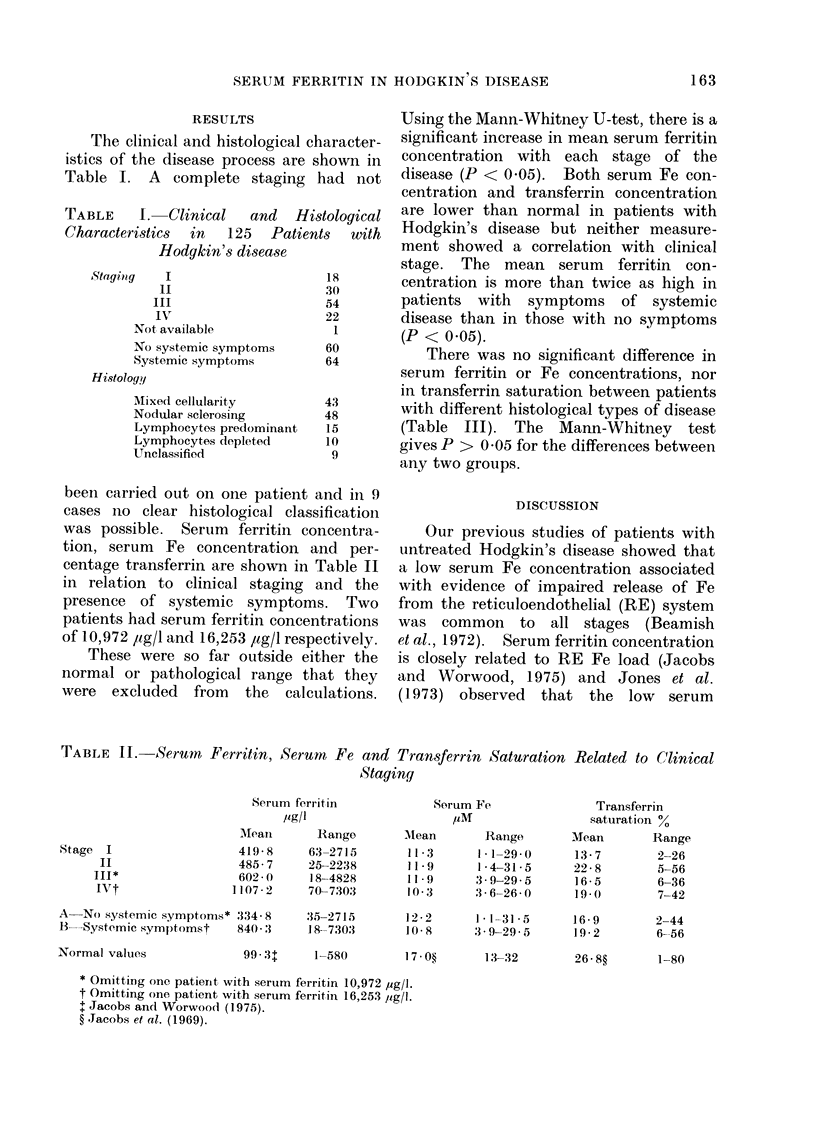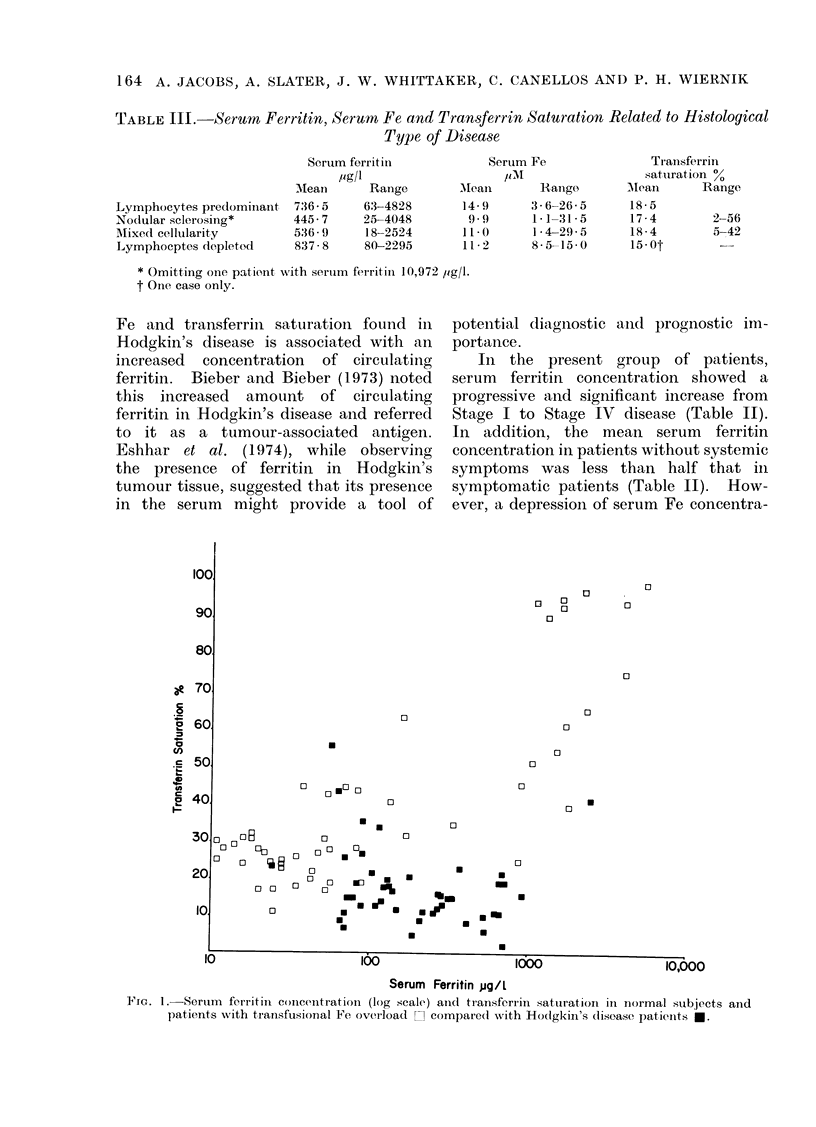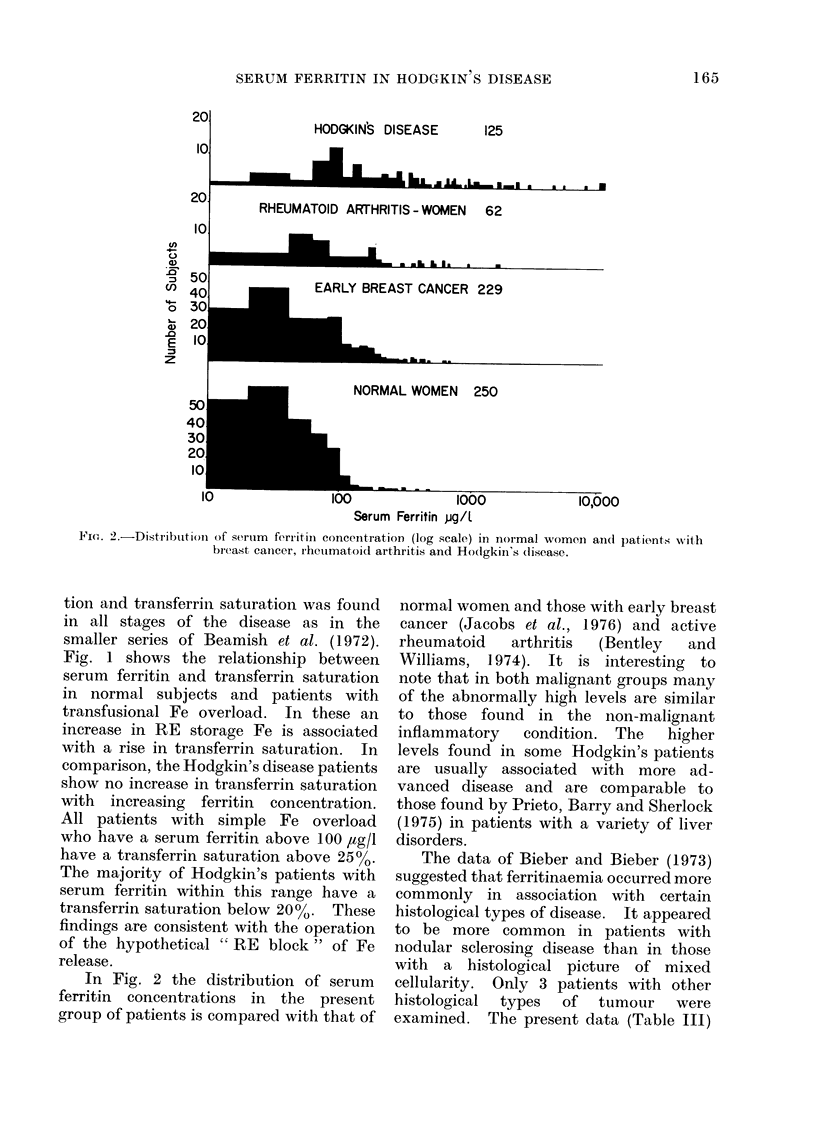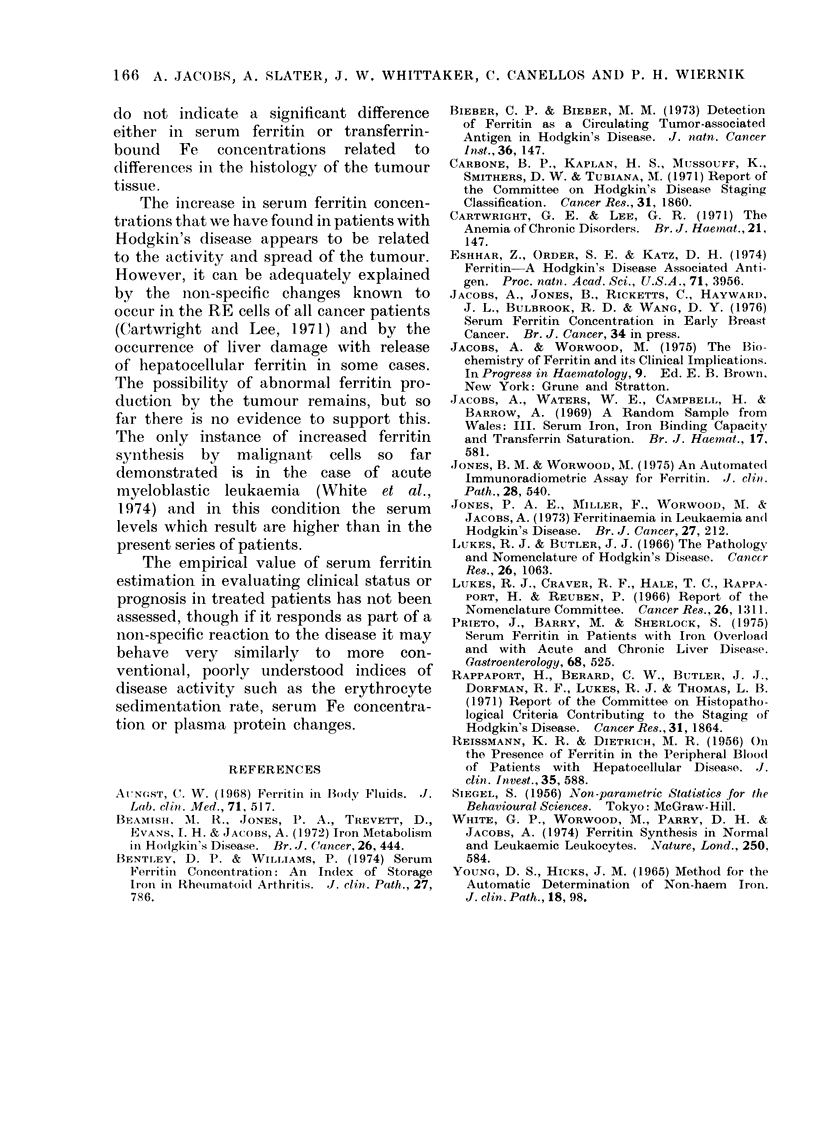# Serum ferritin concentration in untreated Hodgkin's disease.

**DOI:** 10.1038/bjc.1976.138

**Published:** 1976-08

**Authors:** A. Jacobs, A. Slater, J. A. Whittaker, G. Canellos, P. H. Wiernik

## Abstract

Serum ferritin has been estimated in 125 untreated patients with Hodgkin's disease. Increasing concentrations are found at each advancing stage of the disease and high concentrations are found in patients with systemic symptoms. In all cases this is associated with a low serum Fe concentration and reduced transferrin saturation. There is no relationship between serum ferritin concentration and histological type of disease. The findings are compatible with a non-specific response of the reticuloendothelial system to malignancy, producing a secondary disorder of Fe metabolism.


*Br. J. Cancer* (1976) **34**, 162

### SERUM FERRITIN CONCENTRATION IN UNTREATED HODGKIN'S DISEASE

A. JACOBS*, A. SLATER,* J. A. WHITTAKER*, G. CANELLOS† AND P. H. WIERNIK‡

*From * the Department of Haematology, Welsh National School of Medicine, Heath Park, Cardiff, U.K., † the Division of Medical Oncology, Harvard Medical School, Boston, USA, and ‡ the National Cancer Institute, Baltimore Cancer Research Centre, Baltimore, USA.*

Received 19 March 1976 Accepted 26 April 1976

**Summary.—Serum ferritin has been estimated in 125 untreated patients with Hodgkin's disease. Increasing concentrations are found at each advancing stage of the disease and high concentrations are found in patients with systemic symptoms. In all cases this is associated with a low serum Fe concentration and reduced transferrin saturation. There is no relationship between serum ferritin concentration and histological type of disease.**

**The findings are compatible with a non-specific response of the reticuloendothelial system to malignancy, producing a secondary disorder of Fe metabolism.**

FERRITIN has long been recognized as an intracellular Fe storage compound, but its occasional appearance in the circulation has been noted in some malignant states. Reissman and Dietrich (1956), using a relatively crude method, detected ferritin in serum from 6 patients with Hodgkin's disease involving the liver, an observation later confirmed by Aungst (1968) who found ferritin in the serum of 30 patients with Hodgkin's disease. Bieber and Bieber (1973) used a semiquantitative technique to detect ferritin and found it in the serum of 44% of 108 patients with Hodgkin's disease with a particularly high incidence in late-stage nodular sclerosing disease.

A number of workers have shown that ferritin from a variety of tumours displays characteristics similar to that of foetal liver or placenta on isoelectric focusing and have suggested that the production of " carcinofoetal " ferritin may be a characteristic of malignant tissue (Jacobs and Worwood, 1975). Eshhar, Order and Katz (1974) found ferritin to be present in Hodgkin's tissue but characterization of the crystallized protein did not show any differences from normal liver or spleen ferritin. Despite the absence of any detailed information regarding ferritin in Hodgkin's disease it has been suggested that the serum ferritin concentration may be used to monitor disease activity. We have attempted to clarify the relationship between serum ferritin concentration and the stage and type of Hodgkin's disease by analysing the results from 125 untreated patients.

### MATERIALS AND METHODS

Serum samples were obtained from 125 newly diagnosed patients with untreated Hodgkin's disease. In all cases but one, a full staging procedure with laparatomy was carried out and the extent of the disease was categorized according to the classification of the Ann Arbour International Convention (Carbone *et al.*, 1971). Histology of lymph node biopsies was classified according to the classification of Lukes and Butler (1966) as modified at Rye (Lukes *et al.*, 1966) and Ann Arbour (Rappaport *et al.*, 1971). Serum ferritin concentration was estimated by the method of Jones and Worwood (1975) in all 125 cases, serum Fe concentration was measured by a modification of the Young & Hicks (1965) method in 86 cases and transferrin saturation in 49 cases. Standard statistical techniques were used (Siegel, 1956).

### RESULTS

The clinical and histological characteristics of the disease process are shown in Table I. A complete staging had not been carried out on one patient and in 9 cases no clear histological classification was possible. Serum ferritin concentration, serum Fe concentration and percentage transferrin are shown in Table II in relation to clinical staging and the presence of systemic symptoms. Two patients had serum ferritin concentrations of 10,972 μg/l and 16,253 μg/l respectively.

TABLE I.—*Clinical and Histological Characteristics in 125 Patients with Hodgkin's disease*

| | | |
|---|---|---|
| *Staging* | I | 18 |
| | II | 30 |
| | III | 54 |
| | IV | 22 |
| | Not available | 1 |
| | No systemic symptoms | 60 |
| | Systemic symptoms | 64 |
| *Histology* | | |
| | Mixed cellularity | 43 |
| | Nodular sclerosing | 48 |
| | Lymphocytes predominant | 15 |
| | Lymphocytes depleted | 10 |
| | Unclassified | 9 |

These were so far outside either the normal or pathological range that they were excluded from the calculations. Using the Mann-Whitney U-test, there is a significant increase in mean serum ferritin concentration with each stage of the disease ($P < 0{\cdot}05$). Both serum Fe concentration and transferrin concentration are lower than normal in patients with Hodgkin's disease but neither measurement showed a correlation with clinical stage. The mean serum ferritin concentration is more than twice as high in patients with symptoms of systemic disease than in those with no symptoms ($P < 0{\cdot}05$).

There was no significant difference in serum ferritin or Fe concentrations, nor in transferrin saturation between patients with different histological types of disease (Table III). The Mann-Whitney test gives $P > 0{\cdot}05$ for the differences between any two groups.

### DISCUSSION

Our previous studies of patients with untreated Hodgkin's disease showed that a low serum Fe concentration associated with evidence of impaired release of Fe from the reticuloendothelial (RE) system was common to all stages (Beamish *et al.*, 1972). Serum ferritin concentration is closely related to RE Fe load (Jacobs and Worwood, 1975) and Jones *et al.* (1973) observed that the low serum

TABLE II.—*Serum Ferritin, Serum Fe and Transferrin Saturation Related to Clinical Staging*

| | Serum ferritin μg/l | | Serum Fe μM | | Transferrin saturation % | |
|---|---|---|---|---|---|---|
| | Mean | Range | Mean | Range | Mean | Range |
| Stage I | 419·8 | 63–2715 | 11·3 | 1·1–29·0 | 13·7 | 2–26 |
| II | 485·7 | 25–2238 | 11·9 | 1·4–31·5 | 22·8 | 5–56 |
| III* | 602·0 | 18–4828 | 11·9 | 3·9–29·5 | 16·5 | 6–36 |
| IV† | 1107·2 | 70–7303 | 10·3 | 3·6–26·0 | 19·0 | 7–42 |
| A—No systemic symptoms* | 334·8 | 35–2715 | 12·2 | 1·1–31·5 | 16·9 | 2–44 |
| B—Systemic symptoms† | 840·3 | 18–7303 | 10·8 | 3·9–29·5 | 19·2 | 6–56 |
| Normal values | 99·3‡ | 1–580 | 17·0§ | 13–32 | 26·8§ | 1–80 |

* Omitting one patient with serum ferritin 10,972 μg/l.
† Omitting one patient with serum ferritin 16,253 μg/l.
‡ Jacobs and Worwood (1975).
§ Jacobs *et al.* (1969).

TABLE III.—*Serum Ferritin, Serum Fe and Transferrin Saturation Related to Histological Type of Disease*

| | Serum ferritin μg/l | | Serum Fe μM | | Transferrin saturation % | |
|---|---|---|---|---|---|---|
| | Mean | Range | Mean | Range | Mean | Range |
| Lymphocytes predominant | 736·5 | 63–4828 | 14·9 | 3·6–26·5 | 18·5 | |
| Nodular sclerosing* | 445·7 | 25–4048 | 9·9 | 1·1–31·5 | 17·4 | 2–56 |
| Mixed cellularity | 536·9 | 18–2524 | 11·0 | 1·4–29·5 | 18·4 | 5–42 |
| Lymphocptes depleted | 837·8 | 80–2295 | 11·2 | 8·5–15·0 | 15·0† | — |

* Omitting one patient with serum ferritin 10,972 μg/l.

† One case only.

Fe and transferrin saturation found in Hodgkin's disease is associated with an increased concentration of circulating ferritin. Bieber and Bieber (1973) noted this increased amount of circulating ferritin in Hodgkin's disease and referred to it as a tumour-associated antigen. Eshhar *et al.* (1974), while observing the presence of ferritin in Hodgkin's tumour tissue, suggested that its presence in the serum might provide a tool of potential diagnostic and prognostic importance.

In the present group of patients, serum ferritin concentration showed a progressive and significant increase from Stage I to Stage IV disease (Table II). In addition, the mean serum ferritin concentration in patients without systemic symptoms was less than half that in symptomatic patients (Table II). However, a depression of serum Fe concentra-

FIG. 1.—Serum ferritin concentration (log scale) and transferrin saturation in normal subjects and patients with transfusional Fe overload □ compared with Hodgkin's disease patients ■.

FIG. 2.—Distribution of serum ferritin concentration (log scale) in normal women and patients with breast cancer, rheumatoid arthritis and Hodgkin's disease.

tion and transferrin saturation was found in all stages of the disease as in the smaller series of Beamish *et al.* (1972). Fig. 1 shows the relationship between serum ferritin and transferrin saturation in normal subjects and patients with transfusional Fe overload. In these an increase in RE storage Fe is associated with a rise in transferrin saturation. In comparison, the Hodgkin's disease patients show no increase in transferrin saturation with increasing ferritin concentration. All patients with simple Fe overload who have a serum ferritin above 100 $\mu g/l$ have a transferrin saturation above 25%. The majority of Hodgkin's patients with serum ferritin within this range have a transferrin saturation below 20%. These findings are consistent with the operation of the hypothetical "RE block" of Fe release.

In Fig. 2 the distribution of serum ferritin concentrations in the present group of patients is compared with that of normal women and those with early breast cancer (Jacobs *et al.*, 1976) and active rheumatoid arthritis (Bentley and Williams, 1974). It is interesting to note that in both malignant groups many of the abnormally high levels are similar to those found in the non-malignant inflammatory condition. The higher levels found in some Hodgkin's patients are usually associated with more advanced disease and are comparable to those found by Prieto, Barry and Sherlock (1975) in patients with a variety of liver disorders.

The data of Bieber and Bieber (1973) suggested that ferritinaemia occurred more commonly in association with certain histological types of disease. It appeared to be more common in patients with nodular sclerosing disease than in those with a histological picture of mixed cellularity. Only 3 patients with other histological types of tumour were examined. The present data (Table III)

do not indicate a significant difference either in serum ferritin or transferrin-bound Fe concentrations related to differences in the histology of the tumour tissue.

The increase in serum ferritin concentrations that we have found in patients with Hodgkin's disease appears to be related to the activity and spread of the tumour. However, it can be adequately explained by the non-specific changes known to occur in the RE cells of all cancer patients (Cartwright and Lee, 1971) and by the occurrence of liver damage with release of hepatocellular ferritin in some cases. The possibility of abnormal ferritin production by the tumour remains, but so far there is no evidence to support this. The only instance of increased ferritin synthesis by malignant cells so far demonstrated is in the case of acute myeloblastic leukaemia (White *et al.*, 1974) and in this condition the serum levels which result are higher than in the present series of patients.

The empirical value of serum ferritin estimation in evaluating clinical status or prognosis in treated patients has not been assessed, though if it responds as part of a non-specific reaction to the disease it may behave very similarly to more conventional, poorly understood indices of disease activity such as the erythrocyte sedimentation rate, serum Fe concentration or plasma protein changes.

### REFERENCES

AUNGST, C. W. (1968) Ferritin in Body Fluids. *J. Lab. clin. Med.*, **71**, 517.

BEAMISH, M. R., JONES, P. A., TREVETT, D., EVANS, I. H. & JACOBS, A. (1972) Iron Metabolism in Hodgkin's Disease. *Br. J. Cancer*, **26**, 444.

BENTLEY, D. P. & WILLIAMS, P. (1974) Serum Ferritin Concentration: An Index of Storage Iron in Rheumatoid Arthritis. *J. clin. Path.*, **27**, 786.

BIEBER, C. P. & BIEBER, M. M. (1973) Detection of Ferritin as a Circulating Tumor-associated Antigen in Hodgkin's Disease. *J. natn. Cancer Inst.*, **36**, 147.

CARBONE, B. P., KAPLAN, H. S., MUSSOUFF, K., SMITHERS, D. W. & TUBIANA, M. (1971) Report of the Committee on Hodgkin's Disease Staging Classification. *Cancer Res.*, **31**, 1860.

CARTWRIGHT, G. E. & LEE, G. R. (1971) The Anemia of Chronic Disorders. *Br. J. Haemat.*, **21**, 147.

ESHHAR, Z., ORDER, S. E. & KATZ, D. H. (1974) Ferritin—A Hodgkin's Disease Associated Antigen. *Proc. natn. Acad. Sci., U.S.A.*, **71**, 3956.

JACOBS, A., JONES, B., RICKETTS, C., HAYWARD, J. L., BULBROOK, R. D. & WANG, D. Y. (1976) Serum Ferritin Concentration in Early Breast Cancer. *Br. J. Cancer*, **34** in press.

JACOBS, A. & WORWOOD, M. (1975) The Biochemistry of Ferritin and its Clinical Implications. In *Progress in Haematology*, **9**. Ed. E. B. Brown, New York: Grune and Stratton.

JACOBS, A., WATERS, W. E., CAMPBELL, H. & BARROW, A. (1969) A Random Sample from Wales: III. Serum Iron, Iron Binding Capacity and Transferrin Saturation. *Br. J. Haemat.*, **17**, 581.

JONES, B. M. & WORWOOD, M. (1975) An Automated Immunoradiometric Assay for Ferritin. *J. clin. Path.*, **28**, 540.

JONES, P. A. E., MILLER, F., WORWOOD, M. & JACOBS, A. (1973) Ferritinaemia in Leukaemia and Hodgkin's Disease. *Br. J. Cancer*, **27**, 212.

LUKES, R. J. & BUTLER, J. J. (1966) The Pathology and Nomenclature of Hodgkin's Disease. *Cancer Res.*, **26**, 1063.

LUKES, R. J., CRAVER, R. F., HALE, T. C., RAPPAPORT, H. & REUBEN, P. (1966) Report of the Nomenclature Committee. *Cancer Res.*, **26**, 1311.

PRIETO, J., BARRY, M. & SHERLOCK, S. (1975) Serum Ferritin in Patients with Iron Overload and with Acute and Chronic Liver Disease. *Gastroenterology*, **68**, 525.

RAPPAPORT, H., BERARD, C. W., BUTLER, J. J., DORFMAN, R. F., LUKES, R. J. & THOMAS, L. B. (1971) Report of the Committee on Histopathological Criteria Contributing to the Staging of Hodgkin's Disease. *Cancer Res.*, **31**, 1864.

REISSMANN, K. R. & DIETRICH, M. R. (1956) On the Presence of Ferritin in the Peripheral Blood of Patients with Hepatocellular Disease. *J. clin. Invest.*, **35**, 588.

SIEGEL, S. (1956) *Non-parametric Statistics for the Behavioural Sciences.* Tokyo: McGraw-Hill.

WHITE, G. P., WORWOOD, M., PARRY, D. H. & JACOBS, A. (1974) Ferritin Synthesis in Normal and Leukaemic Leukocytes. *Nature, Lond.*, **250**, 584.

YOUNG, D. S., HICKS, J. M. (1965) Method for the Automatic Determination of Non-haem Iron. *J. clin. Path.*, **18**, 98.